# Complex Interactions between Fungal Avirulence Genes and Their Corresponding Plant Resistance Genes and Consequences for Disease Resistance Management

**DOI:** 10.3389/fpls.2017.01072

**Published:** 2017-06-16

**Authors:** Yohann Petit-Houdenot, Isabelle Fudal

**Affiliations:** UMR BIOGER, Institut National De La Recherche Agronomique, AgroParisTech, Université Paris SaclayThiverval-Grignon, France

**Keywords:** avirulence genes, resistance genes, fungal effectors, resistance management, virulence factors

## Abstract

During infection, pathogens secrete an arsenal of molecules, collectively called effectors, key elements of pathogenesis which modulate innate immunity of the plant and facilitate infection. Some of these effectors can be recognized directly or indirectly by resistance (R) proteins from the plant and are then called avirulence (AVR) proteins. This recognition usually triggers defense responses including the hypersensitive response and results in resistance of the plant. *R*—*AVR* gene interactions are frequently exploited in the field to control diseases. Recently, the availability of fungal genomes has accelerated the identification of *AVR* genes in plant pathogenic fungi, including in fungi infecting agronomically important crops. While single *AVR* genes recognized by their corresponding *R* gene were identified, more and more complex interactions between *AVR* and *R* genes are reported (e.g., *AVR* genes recognized by several *R* genes, *R* genes recognizing several *AVR* genes in distinct organisms, one *AVR* gene suppressing recognition of another *AVR* gene by its corresponding *R* gene, two cooperating *R* genes both necessary to recognize an *AVR* gene). These complex interactions were particularly reported in pathosystems showing a long co-evolution with their host plant but could also result from the way agronomic crops were obtained and improved (e.g., through interspecific hybridization or introgression of resistance genes from wild related species into cultivated crops). In this review, we describe some complex *R—AVR* interactions between plants and fungi that were recently reported and discuss their implications for *AVR* gene evolution and *R* gene management.

## Introduction

During infection, pathogens secrete an arsenal of molecules, collectively called effectors, key elements of pathogenesis which modulate innate immunity of the plant and facilitate infection (Oliva et al., 2010). Plants have evolved resistance (*R*) genes encoding R proteins able to recognize, directly or indirectly, some of these effectors [then called avirulence (AVR) proteins]. Recognition of a pathogen AVR protein triggers a set of immune responses grouped under the term Effector-Triggered Immunity (ETI), frequently leading to a rapid localized cell death termed the hypersensitive response (HR) (Jones and Dangl, 2006). Under the selection pressure exerted by *R* genes, pathogens can become virulent through evolution of their *AVR* gene repertoire. Mechanisms leading to virulence include complete deletion, inactivation, or down-regulation of the *AVR* gene, or point mutations allowing recognition to be evaded while maintaining the virulence function of the AVR protein (Jones and Dangl, 2006; Guttman et al., 2014). One class of R proteins corresponds to cell surface LRR-containing R proteins that are anchored to the plasma membrane via a transmembrane (TM) domain and sometimes include an intracellular kinase domain (Receptor-Like Proteins, RLP/Receptor like Kinases, RLK; Yang et al., 2012). The major class of identified R proteins however corresponds to intracellular nucleotide-binding and leucine-rich repeat receptors (NLR). NLR are multi-domain proteins containing a C-terminal leucine-rich repeat (LRR) domain, a central nucleotide-binding (NB) domain and a N-terminal domain often composed of a Toll/interleukin-1 receptor (TIR) or a coiled-coil (CC) domain (Takken and Goverse, 2012). Their multi-domain structure allows R proteins to simultaneously recognize AVR proteins and trigger plant defense reactions. Four models of AVR recognition by R proteins have been proposed and found to co-exist. In the elicitor-receptor model, the R protein directly recognizes its corresponding AVR protein and triggers defense responses (Keen, 1990; Jia et al., 2000; Dodds et al., 2006; Catanzariti et al., 2010; Steinbrenner et al., 2015). In the guard model, the interaction between R and AVR proteins is indirect: the R protein detects modifications of an effector's host target protein, called a “guardee” (Dangl and Jones, 2001). In the decoy model, the R protein detects modifications in a plant protein (called a “decoy”) that mimics the effector target and “traps” the AVR protein (van der Hoorn and Kamoun, 2008). Finally, in the recently proposed integrated decoy model, non-canonical domains mimicking the effector target are integrated into NLRs and play the role of “decoy” (Cesari et al., 2014a; Le Roux et al., 2015, Sarris et al., 2015).

Fungi are the most devastating pathogens of plants, including crops of major economic importance (Fisher et al., 2012). Genetic control is widely used to limit disease development, mainly through the use of major plant *R* genes recognizing fungal *AVR* genes. However, as more and more *R* and *AVR* genes are cloned and their molecular interactions are characterized, an increasing number of complex *R*—*AVR* gene interactions have been identified (Table 1). Such complex *R—AVR* gene interactions potentially result from long co-evolution between plants and pathogens and also from the way agronomic crops were obtained and improved, e.g., through interspecific hybridization or introgression of *R* genes from wild related species. In this review, we highlight some complex *R*—*AVR* gene interactions and discuss how they allow plants to expand pathogen recognition, how pathogens circumvent those plant resistances, and how complex interactions could be managed to improve crop disease resistance.

**Table 1:** Characteristics of fungal avirulence genes and plant resistance genes involved in complex interactions.

**Type of interaction**	**Resistance (*R*) gene (R protein nature, plant species)**	**Use in the fields (durability)**	**Avirulence (*AVR*) gene (fungal species)**	**Interaction R/AVR**	**Involvement in fungal virulence**	**Main molecular mechanisms leading to virulence**	**References**
*AVR* gene recognized by several *R* genes	*Rlm1* (nd, *Brassica napus*)	In the 1990s (overcome in 3 years)	*AvrLm1* (*Leptosphaeria maculans*)	nd	Low (cultivar dependent)	Large deletion (*AvrLm1* and surrounding genomic region)	Rouxel et al., 2003; Gout et al., 2006, 2007; Sprague et al., 2006; Huang et al., 2010; Larkan et al., 2013
	*LepR3* (RLP, *B. napus*)	In 2000 in Australia (overcome in 2 years)					
*AVR* gene recognized by several *R* genes	*Rlm4* (nd, *B. napus*)	Since the 1970's (1999^*a*^)	*AvrLm4-7* (*L. maculans*)	nd	High	One point mutation (no major change of the protein structure)	Huang et al., 2006; Parlange et al., 2009; Daverdin et al., 2012; Balesdent et al., 2015; Blondeau et al., 2015;
	*Rlm7* (nd, *B. napus*)	Since 2005 (beginning of overcome in 2013)	*AvrLm4-7* (*L. maculans*)	nd	High	Inactivating events (deletions, accumulation of mutations)/three point mutations (no major change of the protein structure)	
*AVR* gene recognized by two “cooperating” *R* genes	*Pik-1* and *Pik-2* (NLR, *Oryza sativa*)	Serial deployment (nd)	*AVR-Pik* (*Magnaporthe oryzae*)	Direct with the HMA domain of Pik-1	nd	Point mutations at the interfacing surface involved in Pik/AVR-Pik physical interaction	Yoshida et al., 2009; Kanzaki et al., 2012; Zhai et al., 2014; Maqbool et al., 2015
*AVR* gene recognized by two “cooperating” *R* genes	*RGA4* and *RGA5* (also called *Pi-CO39*, NLR, *O. sativa*)	nd (overcome)	*AVR1-CO39* (*M. oryzae*)	Direct with the HMA domain of RGA5	nd	Deletion	Farman et al., 2002; Okuyama et al., 2011; Cesari et al., 2013; Ribot et al., 2013; de Guillen et al., 2015; Ortiz et al., 2017
	*RGA4* and *RGA5* (also called *Pia*, NLR, *O. sativa*)	nd (overcome)	*AVR-Pia* (*M. oryzae*)	Direct with the HMA domain of RGA5	nd	Deletion/Point mutations (at the hydrophobic surface involved in interaction with RGA5)	
*AVR* gene suppressing recognition of another *AVR* gene	*I-2* (NLR, *Solanum lycopersicum*)	In the 1960s (efficient 20 years in combination with *I*)	*AVR2* (*Fusarium oxysporum* f.sp. *lycopercisium*)	nd	Essential for full virulence	Suppression of *I-2*-mediated recognition by Avr1/Point mutations in *AVR2* (maintaining effector function)	Rep et al., 2005; Houterman et al., 2008, 2009; Catanzariti et al., 2015
	*I-3* (RLK, *S. lycopersicum)*	In the 1980s (nd)	*AVR3* (*F.oxysporum* f.sp. *lycopercisium*)	nd	Essential for full virulence	Suppression of *I-3*-mediated recognition by Avr1	
*AVR* gene suppressing recognition of another *AVR* gene	*Rlm3* (nd, *B. napus*)	nd	*AvrLm3* (*L. maculans*)	nd	nd (conserved in *L. maculans* isolates)	Suppression of *Rlm3*-mediated recognition by AvrLm4-7	Plissonneau et al., 2016, 2017
Bipartite *AVR* gene recognized by one *R* gene	*I-2* (NLR, *S. lycopersicum*)	In the 1960s (efficient 20 years in combination with *I*)	*AVR2* (*F.oxysporum* f.sp. *lycopercisium*)	nd	Essential for full virulence	Point mutations in *AVR2* (maintaining effector function)	Houterman et al., 2009; Ma et al., 2015
			*SIX5* (*F. oxysporum* f.sp. *lycopercisium*)	nd	Essential for full virulence	Conserved in *F. oxysporum* isolates	
*R* gene recognizing several *AVR* genes in distinct organisms	*Cf2* (RLP, *S. lycopersicum*)	In the 1940s (still efficient in combination with other *Cf* genes)	*Avr2 (Cladosporium fulvum*)	Indirect	High	Frameshift mutations	Luderer et al., 2002; Rooney et al., 2005; van Esse et al., 2008; Lozano-Torres et al., 2012; de Wit, 2016
			*Gr-VAP1* (*Globodera rostochiensis*)	Indirect	Essential for infectivity	nd	

*nd, not determined; AVR, avirulence; R, resistance; NLR, Nucleotide-binding and Leucine-rich repeat Receptor; RLP, Receptor-Like Protein; RLK, Receptor-Like Kinase*.

a *The late breakdown time does not reflect durability of Rlm4 since it has been used discontinuously*.

## Avirulence genes recognized by several resistance genes

*AVR* genes recognized by several *R* genes were reported in the pathosystem *Leptosphaeria maculans*/oilseed rape. *L. maculans* is a hemibiotrophic ascomycete responsible for stem canker (Blackleg) of oilseed rape (*Brassica napus*) and is mainly controlled using specific *R* genes often combined with quantitative resistance. To date, 7 *AVR* genes from *L. maculans* have been cloned and all are located in repeat-rich, gene-poor genomic regions (Rouxel and Balesdent, 2017).

*AvrLm1* is recognized by two *R* genes, *Rlm1* and *LepR3*. The two *R* genes are located on different chromosomes and are thus expected to encode different R proteins, although direct evidence is missing to date since only *LepR3* has been cloned (through map-based cloning; Larkan et al., 2013). *AvrLm1* is located as a solo gene in the middle of a 269 kb repeat-rich region. *Rlm1* resistance was deployed in the 1990s and overcome in only 3 years (Rouxel et al., 2003). The main mechanism leading to virulence toward *Rlm1* was a large deletion of *AvrLm1* and its surrounding region (Gout et al., 2007), supporting a limited role of *AvrLm1* in fungal fitness which is cultivar-dependent (Huang et al., 2010). More recently, *AvrLm1* was reported to be recognized by the R protein LepR3, a RLP (Larkan et al., 2013). *LepR3* resistance was rapidly overcome in parts of Australia soon after its introduction (Sprague et al., 2006) as a consequence of the previous use of *Rlm1* cultivars and the deletion of *AvrLm1* in a high proportion of Australian *L. maculans* isolates (Gout et al., 2007).

*AvrLm4-7* is also recognized by two *R* genes, namely *Rlm4* and *Rlm7*. It is unclear whether *Rlm4* and *Rlm7*, which are clustered in the same linkage group but not cloned, are two different genes or two alleles of the same gene (Delourme et al., 2004). In the field, *Rlm4* resistance has been extensively used since the 1970s but is now largely overcome (Rouxel and Balesdent, 2017). The switch to virulence against *Rlm4* was due to a single non-synonymous mutation which does not modify the overall 3-D structure of AvrLm4-7 (Blondeau et al., 2015) and does not affect recognition by *Rlm7* (Parlange et al., 2009). *Rlm7* resistance was deployed in 2004 and then used extensively (e.g., *Rlm7* cultivars comprised 50–70% of the French oilseed crop in 2013; Balesdent et al., 2015). However, the evolution of French *L*. *maculans* populations toward virulence against *Rlm7* was a long process (4% of virulent isolates in 2010, 19% in 2013). The first molecular events leading to virulence toward *Rlm7* mainly corresponded to drastic events (deletion, accumulation of mutations) and also to three amino acid changes without major modification of protein structure (Daverdin et al., 2012; Blondeau et al., 2015). The durability of *Rlm7* resistance may reflect the importance of *AvrLm4-7* for fungal fitness and aggressiveness (Huang et al., 2006) but also the introduction of *Rlm7* into cultivars with high levels of quantitative resistance (Balesdent et al., 2015) and the antagonistic role of *AvrLm4-7* on the *AvrLm3*/*Rlm3* interaction (see section An avirulence gene suppressing recognition of another avirulence gene below). In contrast to the *AvrLm1/Rlm1–LepR3* interaction, the *AvrLm4-7/Rlm4–Rlm7* interaction illustrates that two *R* genes, or possibly two alleles of the same gene, targeting the same *AVR* gene can be deployed successively and be both durable in the field.

## Avirulence genes recognized by two “cooperating” resistance genes

*AVR* genes recognized by two distinct *R* genes that are both necessary for recognition were reported in the *Magnaporthe oryzae*/rice pathosystem. *M. oryzae*, the causal agent of rice blast, is mostly controlled using resistant rice cultivars harboring major *R* genes. Seven *M. oryzae AVR* genes have been cloned (Liu et al., 2013). Interestingly, four of those *AVR* genes (*AVR-Pik, AVR-Pii, AVR1-CO39*, and *AVR-Pia*) are involved in complex interactions, in that two “cooperating” *R* genes are necessary to recognize each AVR (respectively *Pik-1/Pik-2, Pii-1/Pii-2*, and *RGA4/RGA5*; Okuyama et al., 2011; Kanzaki et al., 2012; Cesari et al., 2013; Takagi et al., 2013).

Okuyama et al. (2011) showed that *AVR-Pia* is recognized by two head-to-head *R* genes, *RGA4* and *RGA5*, both being required for resistance. These *R* genes also recognize another *M. oryzae AVR* gene, *AVR1-CO39* (Cesari et al., 2013). In this pair of R proteins, RGA4 acts as constitutively active disease resistance and cell death inducer and is repressed by RGA5 in absence of the pathogen. Direct binding of AVR-Pia or AVR1-CO39 to RGA5 leads to RGA4 de-repression and activation of immune signal transduction (Cesari et al., 2014b). Effector binding to RGA5 occurs in a non-canonical C-terminal domain of RGA5 (called the RATX1/HMA domain) resembling a heavy metal-associated (HMA) domain protein from *Saccharomyces cerevisiae*, thought to function as an integrated decoy domain (Cesari et al., 2013, 2014b; Kroj et al., 2016). The *Pik* locus is also composed of two head-to-head genes separated by a non-coding intergenic region and a HMA domain is present in Pik-1, in this case between the CC and NB domains (Yoshida et al., 2009; Kanzaki et al., 2012). A physical interaction has been demonstrated between AVR-Pik and the HMA domain of Pik-1 (Zhai et al., 2014). Both AVR-Pik and the HMA domain of Pik-1 exhibit amino acid polymorphisms between pathogen isolates and rice cultivars (Yoshida et al., 2009; Kanzaki et al., 2012), located at the interface between Pik-1 and AVR-Pik, meditating their physical interaction and recognition (Maqbool et al., 2015). In *M. oryzae* isolate collections, most are virulent toward *Pia* and *Pi-CO39* and have lost *AVR-Pia* and *AVR1-CO39* (Farman et al., 2002; Cesari et al., 2013). Three isolates virulent toward *Pia* were found to carry an *AVR-Pia* allele with a SNP leading to a non-synonymous substitution, which abolishes interaction with RGA5 and subsequent recognition (Cesari et al., 2013). Recently, Ortiz et al. (2017) found that binding of AVR-Pia to the RATX1 domain of RGA5 involved hydrophobic interactions and that AVR-Pia also interacted with other, as yet undefined, regions of RGA5, increasing the overall effector binding affinity of RGA5 and allowing AVR-Pia recognition and plant defense induction despite the accumulation of point mutations in Avr-Pia and moderate affinity to RATX1. This work highlights the advantage of integrating the decoy domain into the NLR, instead of having the decoy as an independent molecule. Indeed, even if physical interactions between R and AVR proteins favor diversification at the interfacing surfaces, the high resilience of RGA4/RGA5-mediated AVR-Pia recognition to reduction of AVR-Pia-RATX1 interaction strength limits the pathogen's ability to circumvent host recognition. The next step forward would be to fuse other effector targets to NLRs as integrated domains to test whether this can confer increased recognition specificity. These effector targets could themselves be engineered in order to be targeted by a larger panel of effectors and pathogens, such as PBS1 from *A. thaliana*, which cleavage by the bacterial protease AvrPphB is detected by the R protein RPS5, and in which substitution of AvrPphB cleavage site with cleavage sites from other effector proteases extended the recognition specificity of RPS5 to other pathogens (Kim et al., 2016).

## An avirulence gene suppressing recognition of another avirulence gene

Among the proposed roles of pathogen effectors is the suppression of ETI in order to circumvent plant defenses (Jones and Dangl, 2006). In some cases, an effector, which suppresses the AVR activity of another effector, can itself be recognized by an *R* gene, thus allowing mechanistic-based strategies to genetically control plant diseases. Two such cases of *AVR* genes hiding another *AVR* gene have been reported in *L. maculans* and *F. oxysporum*.

*L. maculans* avirulence gene *AvrLm3* is recognized by *Rlm3*. This recognition is suppressed in presence of *AvrLm4-7* which is itself recognized by *Rlm4* and *Rlm7*. Indeed, silencing of *AvrLm4-7* in an isolate virulent toward *Rlm3* allowed recognition by *Rlm3*, and the complementation of an isolate avirulent toward *Rlm3* with *AvrLm4-7* conferred virulence on *Rlm3* cultivars (Plissonneau et al., 2016), confirming the ability of *AvrLm4-7* to suppress *AvrLm3/Rlm3*-mediated resistance and the presence of *AvrLm3* in *L. maculans* populations. *AvrLm3* was recently identified and is located in a telomeric region of the *L. maculans* genome (Plissonneau et al., 2016). The conservation of *AvrLm3* despite its telomeric location suggests an involvement of *AvrLm3* in fungal fitness (Plissonneau et al., 2017). It seems that the main mechanism to acquire virulence toward *Rlm3* was not the deletion of *AvrLm3* but rather the production of an effector, AvrLm4-7, that conceals AvrLm3.

*Fusarium oxysporum f*.sp. *lycopersici* (*Fol*) is a common soil fungus infecting tomato. Several *Fol AVR* genes were identified, including *AVR1* (recognized by *R* genes *I* and *I-1*), *AVR2* (recognized by *I-2*) and *AVR3* (recognized by *I-3*; Rep et al., 2005; Houterman et al., 2008, 2009). *AVR1* is involved in the suppression of *I-3* and *I-2*-mediated recognition of *AVR3* and *AVR2* respectively. Deletion of *AVR1* in an isolate virulent toward *I-2* and *I-3* allowed recognition by *I-3* and *I-2* plants, and the complementation of isolates avirulent toward *I-3* or *I-2* with *AVR1* conferred virulence on *I-3* and *I-2* tomato plants. *AVR3* and *AVR2* were shown to be essential for full virulence of *Fol* on tomato. In agreement, *AVR3* and *AVR2* are never deleted in *Fol* isolates, and no SNP preventing recognition by *I-3* has been identified, while three SNPs preventing recognition by *I-2* without altering virulence of the corresponding isolates were reported (Lievens et al., 2009). In contrast, *AVR1* has no major effect on *Fol* virulence, suggesting that its role is mainly restricted to suppressing *I-2* and *I-3*-mediated recognition (Houterman et al., 2008).

Such interactions offer great opportunities for the genetic control of plant diseases. In tomato, the combination of *I-1*and *I-2*/*I-3* may lead to a durable resistance toward *Fol*, since one *R* gene will be effective against an *AVR* gene important for fungal virulence (*AVR3* or *AVR2*) and another against the suppressor of *I-3*/*I-2*-mediated resistance. The combination of *Rlm7* and *Rlm3* against *L. maculans* could also increase the durability of the two *R* genes in oilseed rape. It is now important to determine whether pyramiding or alternating deployment is the best strategy. Pyramiding the two *R* genes will exert a strong selection pressure on fungal isolates, which could lead to the emergence of isolates virulent toward both resistances. Alternating two resistances in the field combined with a surveillance of *Fol* and *L. maculans* populations would allow counter-selection of virulent isolates.

## A bipartite avirulence gene necessary for recognition by one resistance gene

So far, only a single case of bipartite *AVR* gene/*R* gene interaction has been reported. In *Fol, AVR2*, which triggers *I-2*-mediated recognition and is required for full virulence on susceptible tomato (Houterman et al., 2009), shares its promoter region with *SIX5*, which also encodes a protein secreted in tomato xylem sap. Ma et al. (2015) recently reported that *SIX5* is also required to trigger *I-2*-mediated recognition. Thus, deletion of *SIX5* allows *Fol* to escape *I-2*-mediated resistance, while reintroduction of *SIX5* restores avirulence toward *I-2*, showing that *AVR2* and *SIX5* are both necessary to induce *I-2*-mediated resistance. Avr2 and Six5 physically interact, suggesting that I-2 recognizes the Avr2/Six5 complex. Similar to *AVR2, SIX5* is also present in all *Fol* isolates, and is required for full virulence on tomato (Ma et al., 2015). It is unlikely that specific resistances involved in such bipartite *AVR* gene/*R* gene interactions are more durable, since deletion or point mutation of only one of the *AVR* genes is sufficient to escape recognition by the corresponding *R* gene. Indeed, while no polymorphism was observed in the *SIX5* sequence of isolates virulent toward *I-2*, three point mutations causing single amino acid changes were observed in *AVR2*, allowing *Fol* strains to escape *I-2*-mediated recognition without altering virulence.

## Resistance genes recognizing several avirulence genes in distinct organisms

It has been hypothesized that pathogen effectors target a common set of plant proteins and that plants have evolved surveillance systems to recognize multiple *AVR* genes sharing the same plant target (Mukhtar et al., 2011). Several *R* genes able to recognize distinct pathogens have been reported, which potentially decreases the need for chemical interventions and opens the path to broad-spectrum disease control. A notable example is *Cf2* from tomato, which confers resistance to both the fungal pathogen *Cladosporium fulvum* and the nematode *Globodera rostochiensis* (Rooney et al., 2005; Lozano-Torres et al., 2012).

Several apoplastic effectors of oomycetes, fungi, bacteria and nematodes were reported to target papain-like cysteine proteases (PLCP; Kaschani et al., 2010; Lozano-Torres et al., 2012). Avr2, from the tomato leaf mold agent *C. fulvum*, targets the tomato PLCP Rcr3 and inhibits its activity. Its effector activity on Rcr3 is indirectly recognized by the tomato *R* gene *Cf2*, according to the guard model (Rooney et al., 2005). Cys protease activity profiling showed that Avr2 inhibited multiple extracellular Cys proteases, including Rcr3 and its close relative Pip1, and it was proposed by van der Hoorn and Kamoun (2008) that Pip1 was the operative target of Avr2 and Rcr3 acted as a decoy. Silencing of *Avr2* significantly decreased *C. fulvum* virulence on tomato (van Esse et al., 2008). Interestingly, Rcr3 is also targeted by effectors from other pathogens. For example, an effector of the nematode *G. rostochiensis*, Gr-VAP1, physically interacts with Rcr3 and triggers a *Cf2*-dependent hypersensitive response in tomato (Lozano-Torres et al., 2012). Broad-spectrum resistances exert a strong selection pressure on pathogen populations, potentially leading to them being rapidly overcome. Indeed, even though *Avr2* was demonstrated to be important for virulence, isolates of *C. fulvum* virulent toward *Cf2* were rapidly reported (Luderer et al., 2002). However, *Cf2* is still effective as a result of pyramiding with other specific *R* genes in tomato crops (de Wit, 2016).

## Concluding remarks

While complex interactions between bacterial *AVR* genes and plant *R* genes have been previously discovered and well-studied (Cui et al., 2009; Khan et al., 2016), the characterization of plant/fungal interactions are emerging and show some similarities (cooperating *R* genes, *R* genes recognizing distinct pathogens, *AVR* gene suppressing recognition of another *AVR* gene) but also specificities (bipartite *AVR* gene). Among the *R* genes displaying complex interaction with *AVR* genes, some of the most promising are those conferring broad-spectrum resistances since they guard key components of plant immunity and, as such, target essential effectors. Even if they exert a strong selection pressure on pathogen populations, they may remain effective through pyramiding with other specific or quantitative *R* genes. Another promising strategy to manage durable resistances would be to target antagonistic interactions between *AVR* genes and to combine the corresponding *R* genes in the same cultivars through pyramiding or to sequentially use the *R* genes in rotation. Although antagonistic interactions between *AVR* genes have only been reported twice in plant-fungi pathosystems, they are probably more widely distributed than suspected. Indeed, in cereal powdery mildews it has been suggested that pairs of *AVR* genes and suppressors of *AVR* gene recognition could form the basis of specificity (Bourras et al., 2015, 2016).

## Authors contributions

Both authors reviewed litterature, contributed to writing the manuscript, and approved it for publication.

### Conflict of interest statement

The authors declare that the research was conducted in the absence of any commercial or financial relationships that could be construed as a potential conflict of interest.
